# Detection of Dissolved Lactose Employing an Optofluidic Micro-System

**DOI:** 10.3390/diagnostics2040097

**Published:** 2012-12-06

**Authors:** Emanuel Weber, Franz Keplinger, Michael J. Vellekoop

**Affiliations:** 1 Institute for Microsensors, Actuators and Systems (IMSAS), Microsystems Center Bremen (MCB), University of Bremen, Otto-Hahn-Allee NW1, 28359 Bremen, Germany; E-Mail: mvellekoop@imsas.uni-bremen.de; 2 Institute of Sensor and Actuator Systems, Vienna University of Technology, Gusshausstrasse 27-29, E366, 1040 Vienna, Austria; E-Mail: franz.keplinger@tuwien.ac.at

**Keywords:** lactose detection, non-invasive, label-free, optofluidics, partial total internal reflection

## Abstract

In this work, a novel optofluidic sensor principle is employed for a non-invasive and label-free characterization of lactose containing liquid samples. Especially for medicine and food industry, a simple, fast and accurate determination of the amount of lactose in various products is highly desirable. The presented system exploits the impact of dissolved molecules on the refractive index for sample characterization. On the optofluidic chip, a microfluidic channel filled with the analyte is hit by slightly diverging laser light. The center incident angle of the beam on-chip is set close to the critical angle for total internal reflection. Both the reflected and the transmitted light signals are recorded at the solid-liquid interface. The ratio of those two signals is then used as representative value for the analyte. Using this principle, lactose containing samples were differentiated based on their concentrations at a step size of 10 mmol/L. The use of the signals ratio instead of a single signal approach improves the stability of the system significantly, allowing for higher resolutions to be achieved. Furthermore, the fabrication of the devices in PDMS ensures biocompatibility and provides low absorbance of light in the visible range.

## 1. Introduction

The emergence of lab-on-a-chip devices throughout the last decades has enabled various new sensor devices to be developed. Particularly in medicine and biology, such realizations can be of huge advantage. The reduction in sample volume is crucial for those disciplines. Furthermore, the shortened times required for the analyses hold an enormous potential for medical diagnoses applications. The faster results can be obtained, the higher the possibility of an adequate therapy in case of a disease. In many realizations, small amounts of liquid samples are pumped through those lab-on-a-chip devices and different parameters of the liquid are derived. For the read-out, optical principles hold promising capabilities and are readily implemented. Different internal influences (such as molecule concentration) and external influences (such as temperature) change the optical behavior of the analyte. This change can then be detected and used for sample characterization. Works have been published on micro-flow cytometers [1,2] used for single cell analyses based on light absorbance and fluorescence. In recent works, optical elements on chip were exploited for the characterization of micro-droplets [3,4,5]. Especially, information on the composition of the analyte is highly valuable. Among others, devices based on light deflection [6], interference phenomena [7] and light refraction [8] have been reported for this reason. People suffering from lactose intolerance, for example, are encouraged not to consume high quantities of lactose containing products to avoid unwanted reactions in the body. Therefore, the amount of lactose in various products (solid as well as liquid) is of huge interest for medical purposes and obviously for food industry as well. A simple, fast and robust way to determine the concentration would hence be desirable.

In this work, the impact of dissolved lactose on the refractive index [9] is exploited for the design and realization of a novel sensor system being capable of the above mentioned requirements. Products containing less than 0.1 g lactose per 100 g are considered lactose-free. For liquid products, this equals to a concentration of 3 mmol/L. For lactose intolerant people, the critical lactose concentration in products above which body reactions start to occur is approximately 30 mmol/L. Based on previous work [10], we have set out to elaborate an improved device applying a more suitable fabrication technology to optimize the performance for the detection of lactose in liquid samples at a resolution in the range of those two critical values.

## 2. Theoretical Considerations

The working principle of the device is explained in detail in our previous work [10]. A short explanation of the basics is provided in the following section.

**Figure 1:**
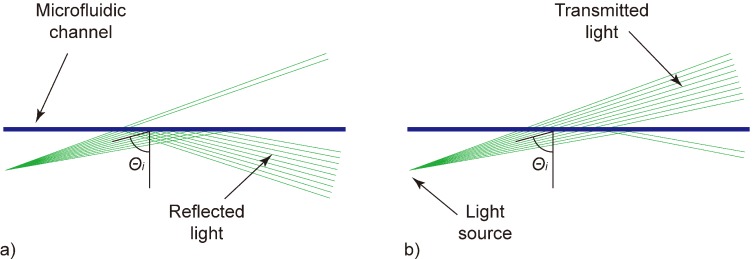
Schematic of the working principle. A straight microfluidic channel (blueline) is hit by slightly diverging laser light (greenlines). (**a**) At samples having a low refractive index, most light rays are totally reflected, while (**b**) at samples with a high refractive index, most light rays are transmitted. The ratio of reflected to transmitted signal is used for sample characterization.

On the optofluidic chip, a straight microfluidic channel filled with the analyte is hit by slightly diverging laser light (Figure 1). The center angle of the incident light (*θ_i_*) is set close to the critical angle for total internal reflection. Due to the intentionally defined divergence of the laser beam, parts of the rays are totally reflected at the solid-liquid interface while other parts are transmitted. The amount of reflected and transmitted light strongly depends on the composition of the analyte. By calculating the ratio of reflected to transmitted light signal, a value representative of the analyte is derived and is used for sample characterization.

**Figure 2:**
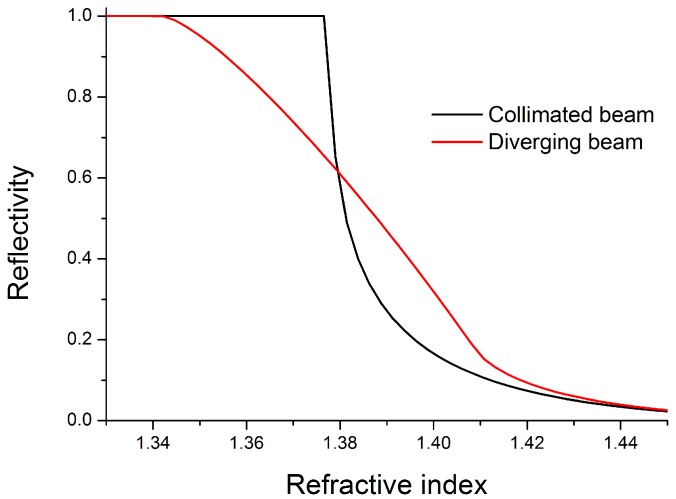
Calculated reflectivities at the solid-liquid interface for different refractive indices. The device with the diverging laser beam shows a linear relation over a large refractive index range, which is advantageous for sample characterization.

At liquids having a relatively low refractive index (low lactose concentration), most light is reflected at the microfluidic channel (Figure 1(a)). With increasing refractive index (increasing lactose concentration), the amount of reflected light decreases while the transmitted part increases simultaneously (Figure 1(b)). Realizations utilizing a fully collimated light beam solely depend on the reflectivity of the analyte according to the Fresnel equations. Such realizations show an inefficient characteristic to changes in the analyte composition. Figure 2 shows the response curves in terms of reflectivity at the solid-liquid interface for two basically different approaches.

The proposed design with a diverging laser beam provides a linear relation between obtained signal ratio and the refractive index of the solution over a huge range. This behavior facilitates a robust application of the principle for an accurate detection of dissolved molecules in the analyte. The theoretical upper end of the feasible working range is defined by the chip material. As long as the index of the chip material exceeds the index of the analyte, which is true for nearly all liquid samples, this method can be applied.

## 3. Materials

Chips were fabricated in polydimethylsiloxane (PDMS) supplied in two separate components (base and curing agent, mixed in a ratio of 10:1; Baltres, Austria). For master device fabrication, dry film resists (Ordyl SY330 and SY317; ElgaEurope, Italy) were used. Ordyl developer was composed of xylene, 2-butoxyethylester, and ethylbenzene (56/30/14, v/v/v; Sigma-Aldrich, USA). Lactose monohydrate (C_12_H_22_O_11_·H_2_O) was purchased from Carl Roth (Germany). Commercially available 1.8%-fat lactose-free milk was used for the final experiments (NÖM, Austria). The external light source was managed by a 531 nm diode pumped solid state laser (20 mW, linear polarization, Roithner LaserTechnik, Austria). Reduced cladding glass fibers (50 µm core diameter, 70 µm cladding diameter, numerical aperture of 0.22; Polymicro Technologies, USA) were used for peripheral light guiding. For the read-out, pre-amplified silicon photodetectors (Thorlabs, USA) were employed. More information about the experimental setup can be found in [10].

## 4. Device Design and Fabrication

**Figure 3:**
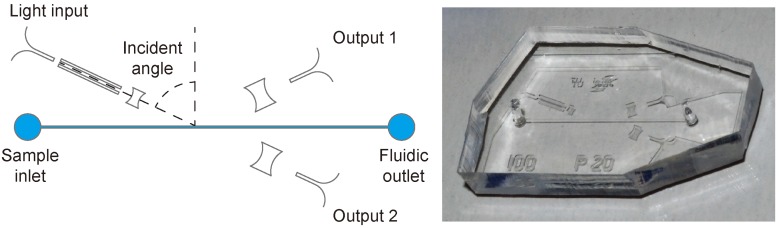
Schematic of the chip design on the left and photograph of one PDMS device on the right. The length of the microfluidic channel is 10.5mm. More dimensions are given in [10] (Reproduced by permission of The Royal Society of Chemistry).

The advantages of the chosen chip material (PDMS) [11,12] are its low optical absorbance in the visible range (380 nm to 780 nm) and its refractive index. Compared with solid materials, the refractive index of PDMS (*n* of 1.41) is relatively low and close to the refractive index of de-ionized water (*n* of 1.33). This adjacency of the refractive indices is beneficial for the device design and hence its performance. Figure 3 shows a schematic of the device design drawn in scale and a photograph of a final PDMS device.

**Figure 4:**
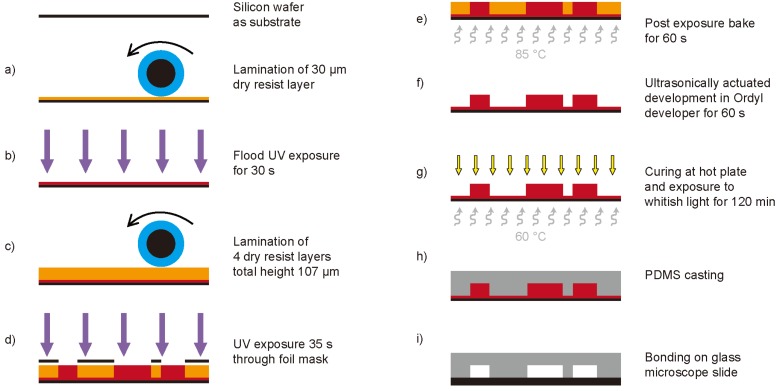
Fabrication process of the micro-chips; (**a**) lamination of a 30µm thick dry resist layer onto a one-sided polished silicon wafer, (**b**) flood UV exposure of the 30µm dry resist layer for 30s to improve the adhesion of following layers, (**c**) consecutive lamination of four layers (one 17 µm and three 30 µm) to reach the final structure height of 107 µm, (**d**) UV exposure (35s) through a high resolution printed foil mask (64,000dpi) to structure the elements, (**e**) post exposure bake for 60s at 85 °C, (**f**) ultrasonically actuated development of the structures in Ordyl developer for 60s, (**g**) curing of the master devices at 60 °C for 120 min under exposure to whitish light, (**h**) PDMS casting, and (**i**) final plasma activated bonding of the PDMS devices onto glass microscope slides.

For the fabrication of the master devices, negative dry film resists were used, Ordyl SY330 and Ordyl SY317 having a thickness of 30 µm and 17 µm, respectively (ElgaEurope, Italy) [3,13]. The fabrication process of the devices is illustrated in Figure 4.

The substrate is a one-sided polished silicon wafer. To enhance the adhesion of small free-standing structures on the substrate, a 30 µm layer of dry resist is laminated onto the wafer (Figure 4(a)). This layer is then flood exposed to UV light (mask aligner MA150, SÜS, Germany) for 30 s (Figure 4(b)). The final height of the microfluidic and optical elements on the device can be defined by the lamination of multiple layers of dry resist on the substrate. For the presented devices a total height of 107 µm was chosen (one 17 µm, and three 30 µm thick layers, Figure 4(c)). This structure height was necessary to allow the applied external glass fibers to be clamped onto the chip (outer diameter of 90 µm). A high-resolution printed polyester foil (64,000 dpi) was employed as mask. Using this mask, the final UV exposure is performed for 35 s (Figure 4(d)). A post exposure bake for 60 s at 85 °C is applied to further enhance the adhesion of the resist (Figure 4(e)). Development is done under ultrasonic agitation for 60 s in Ordyl developer (Figure 4(f)). The finally obtained master devices are then placed on a hot plate (approx. 60 °C) under exposure to whitish light for 120 min to allow curing of the resist (Figure 4(g)). After PDMS casting (Figure 4(h)) the devices are plasma activated and irreversibly bonded on glass microscope slides to seal the microfluidic channels (Figure 4(i)).

This technique allows fabrication of master devices within a few hours. This is of huge advantage especially for proof of concept studies.

## 5. Results and Discussions

Before fabricating the devices, ray-tracing simulations were carried out to evaluate the performance of the designs (ZEMAX, USA). The working ranges were optimized for lactose concentrations between 0 mol/L up to 0.5 mol/L corresponding to refractive indices of 1.333 and 1.358 [9], respectively.

### 5.1. First Characterization

**Figure 5:**
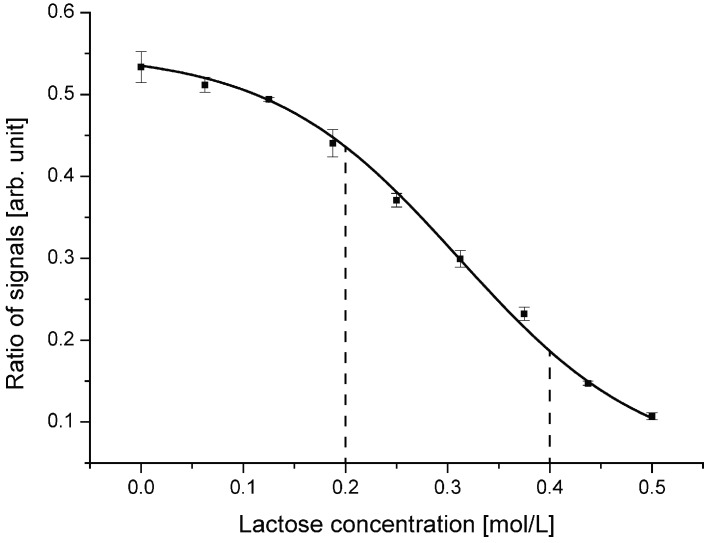
Ratio of reflected to transmitted light signal for the device with an incident angle of 72.5 °. Highest sensitivity is obtained between 0.2 mol/L and 0.4 mol/L and defines the optimal working range.

The first investigated device was designed with a center incident angle of 72.5 °. The divergence of the laser beam was set to approximately ±3 °. This divergence is defined by an integrated air micro-lens on-chip. To characterize the performance of the device, lactose was dissolved in de-ionized (DI) water at concentrations between 0 mol/L and 0.5 mol/L. Those solutions were then analyzed in the optofluidic chip one after another. Figure 5 illustrates the obtained ratios of reflected to transmitted light plotted over the concentration. The results show the same tendency as anticipated by the simulations. A linear working range of the fitted sigmoid function over a defined region is evident.

The gradient of the curve represents the sensitivity of the device at the given lactose concentration. Highest sensitivity is obtained in the linear region between 0.2 mol/L and 0.4 mol/L. At lower concentrations the curve saturates. At those values, the amount of reflected and transmitted light does not change significantly anymore. Nearly all of the incident light rays are totally reflected. At higher concentrations, almost no light ray experiences total internal reflection anymore. There still is a change in the reflected and transmitted signal according to the Fresnel equations. Anyway, the impact of changes in the refractive index on the output decreases, which degrades the performance of the device. To obtain higher sensitivity for other concentration regions, the center incident angle of the light beam has to be adjusted.

### 5.2. Device Optimization for Low Lactose Concentrations

**Figure 6:**
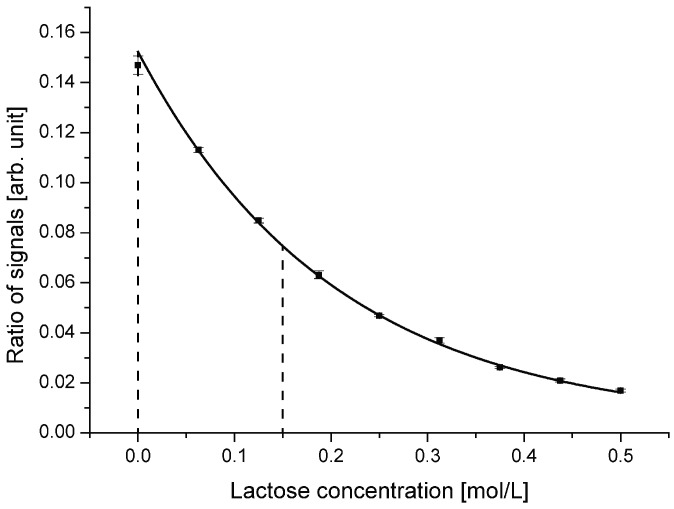
Ratio of reflected to transmitted light signal for the device with an incident angle of 70.0 °. Compared with the device with an incident angle of 72.5 °, the working range is shifted to lower lactose concentrations. The optimal working range is defined between 0 mol/L and 0.150 mol/L.

To optimize the performance of the sensor system for lowest lactose concentration and to obtain the smallest value for the detection limit, a design with a reduced incident angle was developed. At reduced incident angle, the working range and with that the region of highest sensitivity will be shifted to smaller values. The optimized device was designed with an incident angle of 70.0 °. Figure 6 depicts the ratio of reflected to transmitted light signal for this design.

Compared with the first device, a clear shift of the working range towards lower lactose concentrations is evident. The gradient of the curve is highest around 0 mol/L, meaning pure DI water. For concentrations exceeding 0.15 mol/L, the sensitivity decreases rapidly, which indicates the end of the optimal working range. In addition to changing the center incident angle to 70.0 °, also the light coupling region was optimized. By fine tuning the external fiber coupling, an increased coupling coefficient and with that higher optical power on-chip was achieved while using the same light source.

**Figure 7:**
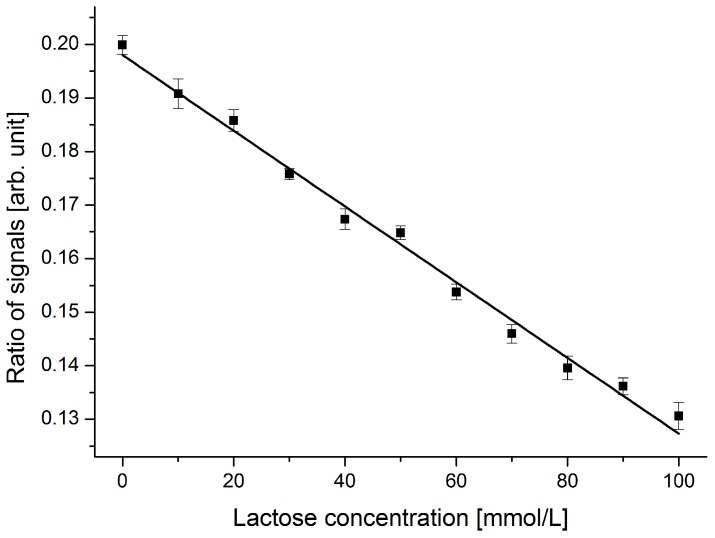
Response of the sensor device to dissolved lactose in DI water in the range from 0 to 100 mmol/L. Samples with different lactose concentrations in a step size of 10 mmol/L can clearly be discriminated. Concentration errors and laser power stability issues explain the small deviations from the fitted linear curve.

Considering the standard deviation obtained for the device with an incident angle of 70.0 °, a smallest detectable change of below 10 mmol/L lactose was anticipated. To demonstrate this capability, lactose concentrations of 0 mol/L up to 100 mmol/L with a step size of 10 mmol/L were analyzed in the device. The obtained results are shown in Figure 7.

For this analysis, four series of measurements were performed on different samples containing the same amounts of dissolved lactose. The variation in the sensor output for a given analyte was in the noise level of the optical setup and hence not detectable. With the optimized device, a discrimination of samples containing different lactose concentrations in a step size of 10 mmol/L is achieved over the entire working range. This step size is equivalent to a difference in refractive index of two consecutive samples of 5 × 10^−4^. Imperfections in sample preparation (*i.e.*, concentration errors) and instable laser conditions are responsible for the small deviations from the fitted linear curve.

### 5.3. Experiments on Off-the-Shelf Lactose-Free Milk

**Figure 8:**
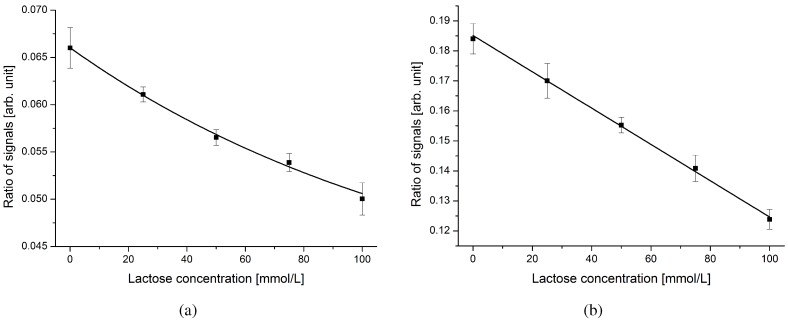
Sensor output of two different devices for complex analytes. Known concentrations of lactose were added to untreated lactose-free milk. The high optical absorbance of milk explains the increased standard deviations. (**a**) The device with an incident angle of 70.0 ° is operating outside the linear region. (**b**) The device with an incident angle of 72.5 ° allows operation in the linear range. A univocal discrimination of samples in a step size of below 25 mmol/L is feasible.

For a final evaluation of the performance of the system on a complex mixture, untreated lactose-free milk was taken as the buffer solution. Without any cleaning or washing steps, the lactose-free milk was spiked with known concentrations of lactose. Aliquots were then analyzed in two different devices. Figure 8 depicts the obtained results.

Four series of measurements were performed on both devices. In the diagrams, an overall increase of the standard deviations is evident. The main reason is the reduced transparency of milk compared with DI water at the employed wavelength (531 nm). The transmitted light signal experiences high absorbance and its impact on the ratio of the reflected-to-transmitted light signal decreases. Reducing the width of the analysis channel would minimize this effect. Furthermore, ingredients of the lactose-free milk (e.g., different types of sugar, salts) increase the overall refractive index of the buffer solution. At increased refractive indices, the device with an incident angle of 70.0 ° (Figure 8(a)) is working outside the linear region. Using a device optimized for higher refractive indices (incident angle of 72.5 °, Figure 8(b)) allows operation in the linear region again. Samples containing different concentrations of dissolved lactose in a step size of below 25 mmol/L can clearly be distinguished in the range of 0 to 100 mmol/L.

## 6. Conclusions

Using the presented optofluidic sensor system, lactose concentrations in a step size of 10 mmol/L were successfully discriminated with DI water as buffer solution. Experiments on lactose-free milk proved the applicability of the system even for complex mixtures. A resolution of below 25 mmol/L in the whole range of 0 up to 100 mmol/L was achieved. The analysis sequence is non-invasive and label-free, requiring minimal manual interventions. In a simple flow-through experiment, the analytes were characterized in real time. If converted to refractive index units, a resolution of 5 × 10^−4^ is achieved, which is comparable to conventional bench-top refractometers. Especially the simplicity in design allows for a rapid and cost-effective production of the device. By tuning the incident angle and the parameters of the integrated optical components, a design can be optimized for different specifications and integrated on-chip together with other microfluidic elements.

Due to its robustness, simplicity and flexibility in integration, this optofluidic sensor system has the potential for the design and realization of on-chip analysis systems for various biologically or medically relevant sample solutions.
